# Ivabradine-Stimulated Microvesicle Release Induces Cardiac Protection against Acute Myocardial Infarction

**DOI:** 10.3390/ijms21186566

**Published:** 2020-09-08

**Authors:** Rafael Ramirez-Carracedo, Laura Tesoro, Ignacio Hernandez, Javier Diez-Mata, Laura Botana, Marta Saura, Marcelo Sanmartin, Jose Luis Zamorano, Carlos Zaragoza

**Affiliations:** 1 Cardiology Department, Universidad Francisco de Vitoria/Hospital Ramón y Cajal Research Unit (IRYCIS), 28223 Madrid, Spain; rrcarracedo@hotmail.com (R.R.-C.); lauratesoro4@hotmail.com (L.T.); naxete1992@gmail.com (I.H.); jdiezmata@gmail.com (J.D.-M.); laura_209g@hotmail.com (L.B.); 2 CIBER de Enfermedades Cardiovasculares (CIBERCV), Instituto de Salud Carlos III (ISCIII), 28029 Madrid, Spain; marta.saura@uah.es (M.S.); msanfer@me.com (M.S.); zamorano@secardiologia.es (J.L.Z.); 3 Systems Biology Department, Facultad de Medicina Universidad de Alcalá, IRYCIS, 28772 Alcala de Henares, Spain; 4 Cardiology Department, IRYCIS, 28034 Madrid, Spain

**Keywords:** acute myocardial infarction, ischemia/reperfusion, microvesicles, ivabradine, EMMPRIN

## Abstract

Ivabradine can reduce heart rate through inhibition of the current I(*f*) by still unexplored mechanisms. In a porcine model of ischemia reperfusion (IR), we found that treatment with 0.3 mg/kg Ivabradine increased plasma release of microvesicles (MVs) over Placebo, as detected by flow cytometry of plasma isolated from pigs 7 days after IR, in which a tenfold increase of Extracellular Matrix Metalloproteinase Inducer (EMMPRIN) containing (both high and low-glycosylated) MVs, was detected in response to Ivabradine. The source of MVs was investigated, finding a 37% decrease of CD31+ endothelial cell derived MVs, while CD41+ platelet MVs remained unchanged. By contrast, Ivabradine induced the release of HCN4+ (mostly cardiac) MVs. While no differences respect to EMMPRIN as a cargo component were found in endothelial and platelet derived MVs, Ivabradine induced a significant release of EMMPRIN+/HCN4+ MVs by day 7 after IR. To test the role of EMMPRIN+ cardiac MVs (EMCMV), H9c2 cell monolayers were incubated for 24 h with 10^7^ EMCMVs, reducing apoptosis, and increasing 2 times cell proliferation and 1.5 times cell migration. The in vivo contribution of Ivabradine-induced plasma MVs was also tested, in which 10^8^ MVs isolated from the plasma of pigs treated with Ivabradine or Placebo 7 days after IR, were injected in pigs under IR, finding a significant cardiac protection by increasing left ventricle ejection fraction and a significant reduction of the necrotic area. In conclusion ivabradine induces cardiac protection by increasing at least the release of EMMPRIN containing cardiac microvesicles.

## 1. Introduction

Coronary artery disease (CAD) and chronic heart failure (CHF) are two leading causes of death worldwide. In attempting to restore adequate tissue perfusion, alterations on blood pressure and heart rate seriously increases myocardial oxygen [1] and compromises ventricular filling efficiency by shortening diastolic duration [2]. Hence, reduction of HR could represent a lifesaving strategy for these patients.

Beta-blocker administration was efficiently proved to reduce heart rate, however, undesired side effects like negative inotropic stimulation, highlighted the need of finding new pharmacological alternatives. Several clinical trials have proved the benefits of using the Funny I(*f*) current inhibitor Ivabradine for the treatment of CAD and CHD, including the SHIFT study (Systolic Heart failure treatment with the If inhibitor ivabradine Trial), in which Ivabradine has been shown to reduce re-hospitalization and mortality rates in patients with CHF and reduced left ventricular ejection fraction (LVEF) [3]. In addition, the contribution of Ivabradine in acute heart failure (AHF) was recently reported, confirming the effectiveness against dobutamine-induced tachycardia, and improving the hemodynamic parameters immediately after Acute Myocardial Infarction (AMI) and long-term [4,5], but the underlying molecular mechanisms are yet to be investigated.

Ivabradine was designed to bind to the hyperpolarization activated cyclic nucleotide gated potassium channel 4 (HCN4), decreasing heart rate and with no undesired effects on blood pressure, excessive e oxygen consumption, and inotropic effects. However, recent research have demonstrated that in addition to blocking HCN channels, new evidences have emerged so far, suggesting alternative molecular targets of Ivabradine. Such is the case of cardiac protection against Coxackievirus infection through inhibition of MAPKinase activation [6], and the new mechanism elicited by Ivabradine to prevent cardiogenic shock-induced extracellular matrix degradation through inhibition of Extracellular Matrix Metalloproteinase Inducer (EMMPRIN), a major pro-inflammatory effector implicated in cardiac necrosis through extracellular matrix metalloproteinase activation (In Press), suggesting a major role of Ivabradine against inflammation.

Extracellular vesicles (EVs) have emerged as one of the most significant inflammatory signaling responsive elements in the recent years [7]. Their capacity to transport nucleic acids, proteins and to participate in molecular pathways make EVs the subject of intense study as potential diagnostic and/or therapeutic tools, since EV presence or absence can often make a difference between cell proliferation or cell death [8,9,10].

Several types of EVs have been described so far. As defined by their biogenesis, we may find exosomes (50–100 nm), shed from multi-vesicular bodies; microvesicles (100–1000 nm), shed from the plasma membrane; and apoptotic blebs (1000–5000 nm), formed as result of apoptosis. Microvesicles (MVs) are medium-size vesicles that shed directly from the plasma membrane, and, in the heart, depending on their cargo composition, EVs shed from cardiomyocytes after ischemia are known to promote both proliferative and apoptotic signals [8,10].

Proteolysis of the extracellular matrix leads to cardiac necrosis during the onset and progression of myocardial infarction. Among others, Extracellular Matrix Metalloproteases (MMPS), and the MMP inducer EMMPRIN, play a significant role in myocardial infarction [11]. However, MMPs and EMMPRIN are also cargo components of many MVs involved in the migration and proliferation of several cancer and epithelial cells [12,13,14,15]. We hypothesize that the better outcomes experienced by patients in response to Ivabradine could be attributed, at least in part, to its effect on MV release, preventing cardiac cell death.

## 2. Results

### 2.1. Ivabradine Increases Microvesicle Release after Ischemia Reperfusion (IR)

**Figure 1:**
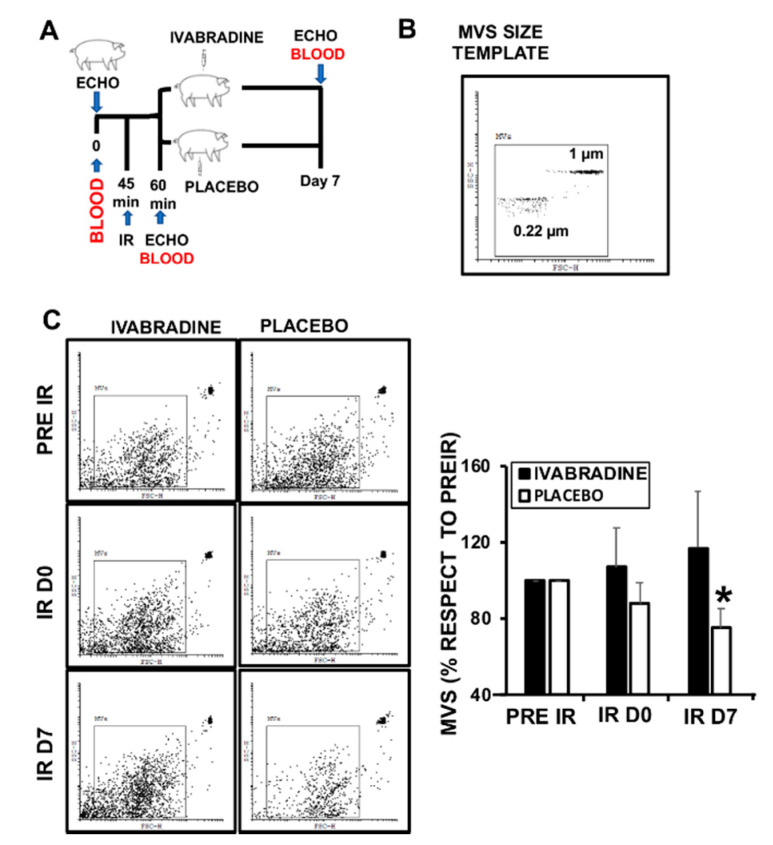
Ivabradine regulates the release of total microvesicles (MVs) after ischemia reperfusion (IR). (**A**) Schematic representation of the procedure. (**B**) MVs size template. (**C**) Flow cytometry detection of plasma MVs at different times after IR (*n* = 5 pigs/group, mean ± SD; * *p* < 0.002 IR D7 Ivabradine vs. Placebo).

Animals subjected to IR and treated with 0.3 mg/kg Ivabradine (Figure 1A), had lower heart rate, and better stroke and left ventricle ejection fraction, as previously described [4,5]. The recent findings on blood released microvesicles (MVs) in response to acute myocardial infarction [10], led us to investigate whether Ivabradine may have an effect in the amount and/or composition of MV release, with the criteria of restricting events gated between 0.22 µm and 1 µm (Figure 1B). Total MVs isolated from the plasma of pigs subjected to IR were collected at different times after surgery (pre-IR, D0 and D7 post-IR), showing no significant differences in total MV content, except for plasma MVs collected 7 days post-IR (IR D7) from the Ivabradine group (Figure 1C).

### 2.2. Ivabradine-Induced Microvesicles Improve Cardiac Function in Pigs Subjected to IR

**Figure 2:**
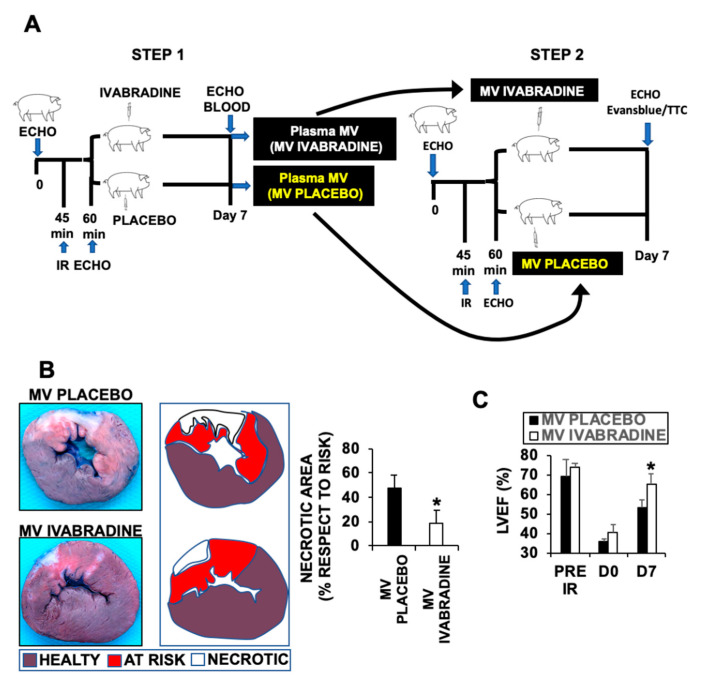
Ivabradine released MVs from pigs 7 days after IR, induce cardiac protection against myocardial infarction. (**A**) Schematic representation of the procedure. (**B**) Evans blue/TTC staining of 1 cm heart sections from pigs who underwent IR and were injected with MVs from Placebo or Ivabradine injected pig, showing the necrotic area (white) respect to the area at risk (red) (*n* = 3 pigs/group. * *p* < 0.05 MV Placebo vs. MV Ivabradine). (**C**) Left ventricle ejection fraction from the same pigs as in (B), estimated before IR, and at times 0 (D0) and 7 days (D7) post-IR (* *p* < 0.05 D7 MV Placebo vs. MV Ivabradine).

To test whether MVs may have an impact on cardiac function against myocardial infarction, we isolated plasma MVs by day 7 after myocardial infarction from pigs treated with 0.3 mg/kg Ivabradine or Placebo (Figure 2A). Injection of 1 mL/kg (10^8^) MVs into pigs subjected to IR, revealed a significant improvement of cardiac function after 7 days of IR, as detected by a significant increase of left ventricle ejection fraction of hearts from pigs injected with MVs isolated from animals who underwent IR and treated with Ivabradine respect to the Placebo MVs (Figure 2B). The improvement of cardiac function corresponded an important reduction of cardiac necrosis, as shown by Evansblue/TTC staining of cardiac sections isolated 7 days after IR (Figure 2C).

### 2.3. Ivabradine Induces the Release of Cardiac Circulating Microvesicles in Pigs Subjected to IR

**Figure 3:**
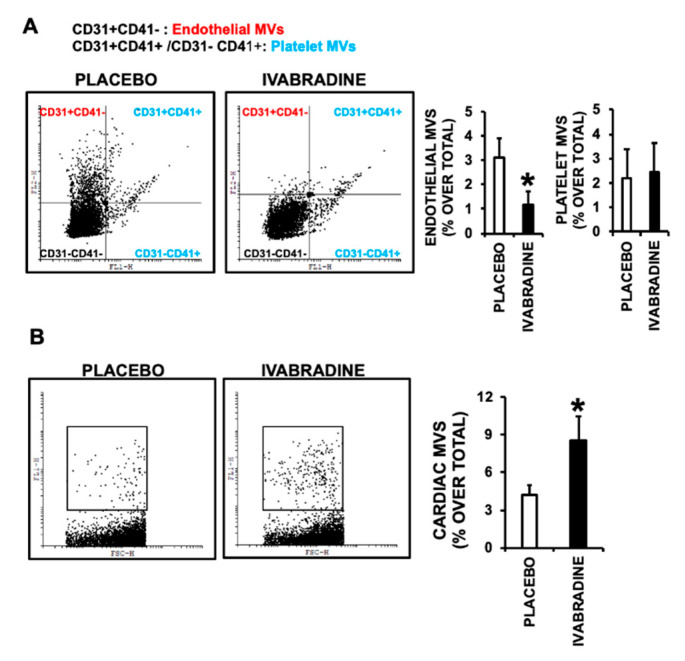
Ivabradine regulates the release of endothelial and cardiac MVs after IR. **A**. Plasma MVs of Endothelial (EMVs) and Platelet (PMVs) origin isolated 7 days after IR. Upper-left quadrant shows EMVs and Upper-right quadrant shows PMVs. (*n* = 5 pigs/group, mean ± SD; * *p* < 0.001 ECMVs Ivabradine vs. Placebo) **B**. Cardiac origin (CMVs) isolated 7 days after CS (*n* = 5 pigs/group, mean ± SD; * *p* < 0.001 CMVs Ivabradine vs. Placebo).

With the aid of specific antibodies, we quantified the release of CD31+ endothelial MVs, CD31+/CD41+ platelet MVs (since CD31 is also expressed in platelets), and HCN4+ derived MVs by flow cytometry. We show that Ivabradine reduced the release of endothelial-CD31+/CD41-derived MVs, while the content of Platelet CD31+/CD41+ MVs remained unchanged (Figure 3A). By contrast, HCN4+ MVs (mostly cardiac-specific) were increased by 50% in the plasma of pigs treated with Ivabradine (Figure 3B).

### 2.4. Ivabradine Increases EMMPRIN-Containing Microvesicles after IR

**Figure 4:**
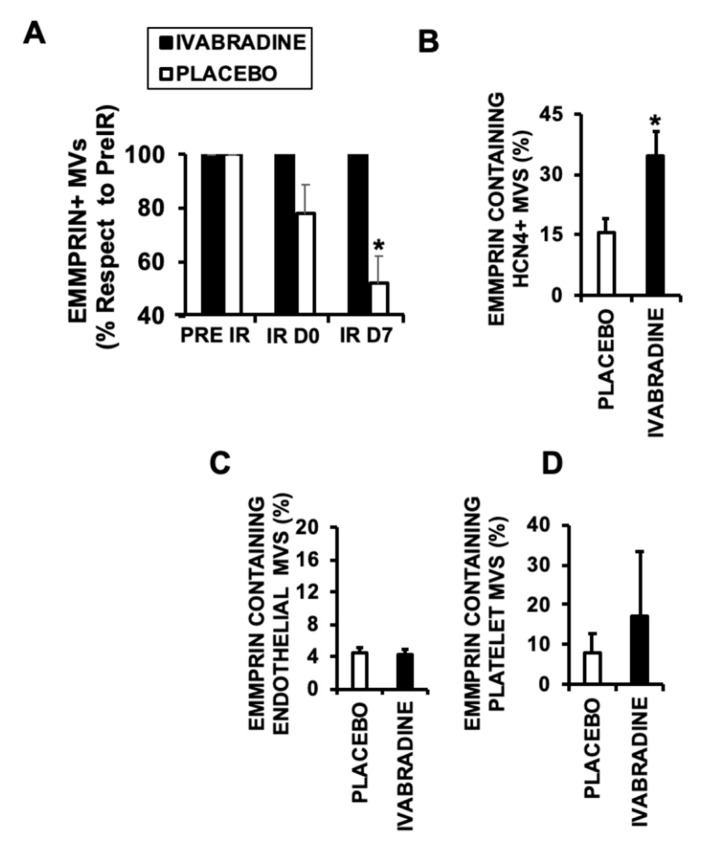
Ivabradine induces the release of EMMPRIN-containing MVs after IR. (**A**). Flow cytometry detection of plasma containing EMMPRIN+ MVs at different times after IR (*n* = 5 pigs/group, mean ± SD; * *p* < 0.001 IR D7 Ivabradine vs. placebo). (**B**). Flow cytometry detection of plasma HCN4+/EMMPRIN+ MVs by day 7 after IR (*n* = 5 pigs/group, mean ± SD; * *p* < 0.05 Ivabradine vs. placebo). (**C**,**D**). Flow cytometry detection of plasma CD31+/CD41−/EMMPRIN+ (endothelial), and CD31+/CD41+/EMMPRIN+ (platelet) MVs, respectively, by day 7 after IR.

As mentioned above, proteolysis is a leading cause of cardiac necrosis during the onset and progression of myocardial infarction. We previously found that IR induces the expression of several proteolytic degrading enzymes, including matrix metalloproteinases [16], and the Extracellular Matrix Metalloproteinase Inducer EMMPRIN (CD147, Basigin), a glycoprotein when highly-glycosylated (HG-EMMPRIN), may activate several MMPs, including MMP-9 and MMP-13 [16,17,18]. MMPs and EMMPRIN are usually cargo components of many MVs involved in the migration of several cancer and epithelial cells [12,13,14,15]. We found that IR reduced the release EMMPRIN-containing MVs by more than 70%, and that was reversed in response to Ivabradine (Figure 4A). More cardiac HCN4+/EMMPRIN+ MVs were found in response to Ivabradine (Figure 4B), while it did not change the levels of endothelial CD31+CD41−/EMMPRIN+ or platelet CD31+/CD41+/EMMPRIN+ MVs (Figure 4C,D).

### 2.5. Ivabradine-Induced Cardiac MVs Promote Cardiac Cell Migration, Proliferation, and Prevent Apoptotic Cell Death

**Figure 5:**
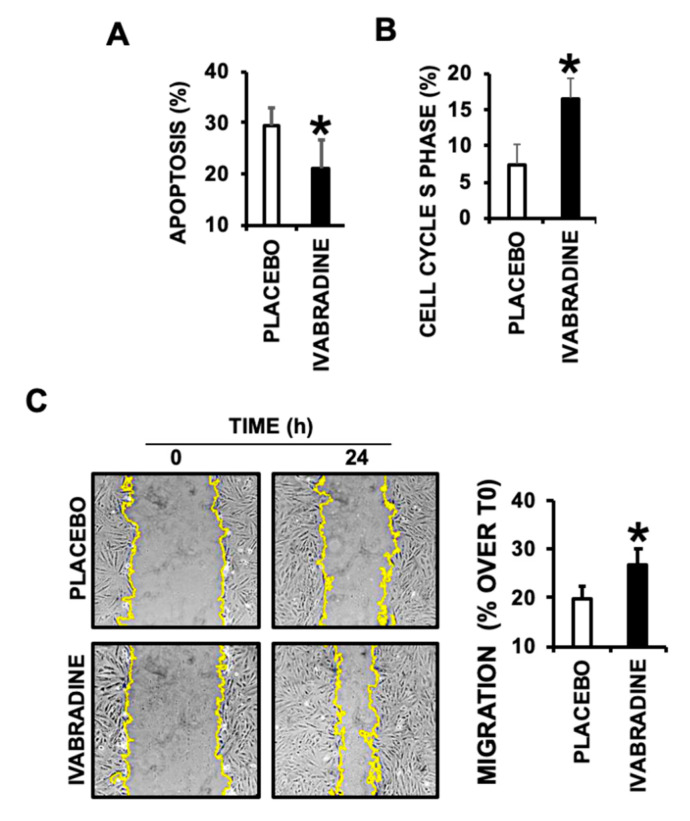
EMMPRIN+ cardiac MVs prevent cardiac cell apoptosis and induce cell proliferation and migration of H9c2 cell monolayers. (**A**) Flow cytometry detection of apoptosis in H9c2 incubated with 10^7^ EMMPRIN+ cardiac MVs from Placebo or Ivabradine treated pigs 7 days after IR (*n* = 3, * *p* < 0.05 Placebo vs. Ivabradine). (**B**) S-Phase detection by flow cytometry in the same cells as in A (*n* = 3, * *p* < 0.05 Placebo vs. Ivabradine). (**C**) Wound healing assay in H9c2 cells incubated with 10^7^ EMMPRIN+ cardiac MVs from Placebo or Ivabradine treated pigs, isolated 7 days after IR (*n* = 3, * *p* < 0.05 Placebo vs. Ivabradine).

To test the contribution of Ivabradine-induced cardiac MVs, 24-h serum deprived cultured cardiac derived H9c2 cells were incubated for 24 h with 10^7^ Ivabradine or Placebo MVs, finding a significant reduction of apoptosis (Figure 5A), and a cell cycle S-phase increase, indicative of cell proliferation (Figure 5B). Furthermore, a wound healing assay performed in H9c2 cell monolayers, also revealed a positive effect in cell migration, consistently with EMMPRIN-induced MMP activation, in response to Ivabradine derived MVs (Figure 5C).

## 3. Discussion

In the current work, we investigated a new mechanism of cardiac protection induced by ivabradine against cardiac ischemia/reperfusion damage. In a porcine model of IR, we found that Ivabradine improved cardiac function after 7 days of reperfusion, in which a significant amount of plasma released MVs was detected. Intracardial release of extracellular vesicles, characterized as exosomes and microvesicles, are induced in response to acute myocardial infarction [19,20], suggesting that plasma circulating microvesicles are markers of progression of infarction [19,20,21,22,23]. Injection of Ivabradine-induced Plasma MVs isolated 7 days after IR mimicked the effect of Ivabradine in pigs subjected to acute myocardial infarction, suggesting that at least, Ivabradine may induce cardiac protection by releasing cardiac specific MVs. While Ivabradine had no effect on platelet MVs and inhibited the release of endothelial MVs, cardiac derived MVs were significantly increased after 7 days of reperfusion. We also evaluated the presence of EMMPRIN in Ivabradine induced MVs, since Extracellular matrix proteolysis is a major disadvantage against IR, and MMPs and EMMPRIN were important cargo components in MVs involved in cell proliferation and migration. We found that EMMPRIN containing cardiac MVs inhibited apoptosis and promoted cell migration and proliferation of H9c2 cardiac cells.

Circulating endothelial-derived MVs are considered good markers for cardiovascular diseases. The levels of these MVs are higher in inflammatory-related diseases, as atherosclerosis, diabetes, or autoimmune pathologies. In addition, endothelial-derived MV levels are elevated in hypertensive patients and are used as markers to monitor myocardial infarction in the long-term [9,24]. Abbas et al. revealed that those MVs induce premature coronary artery endothelial cell aging and thrombogenicity, suggesting that targeting the shedding and bioavailability may represent a novel therapeutic strategy to limit endothelial dysfunction-derived complications post infarction [24]. In according with these finding, we describe that Ivabradine reduced the release of endothelial-derived MVs 7 days after IR and, therefore, may serve as therapeutic tool against the negative effects of endothelial dysfunction in the heart after myocardial infarction.

Cardiac injury induces the release of circulating EVs from all cardiovascular cell types, including endothelial cells, platelets, and cardiac myocytes, and have been not only proposed as reliable diagnostic targets but also as therapeutic promising tools [25]. Here, we find, for the first time, that Ivabradine derived cardiac MVs induce cardiac protection against IR.

The expression of EMMPRIN is induced in cardiac myocytes and lymphocytes as part of the inflammatory response that happens in the absence of blood perfusion in the necrotic areas of the heart, and therefore strongly contributes to cardiac myocyte cell death by inducing MMP-mediated extracellular matrix degradation. We and others found that targeting EMMPRIN leads to cardiac protection by preserving cardiac function and extracellular matrix integrity [17,18]. Hence, our results point towards a new cardioprotective effect of Ivabradine, by releasing specific circulating MVs. Whether EMMPRIN is responsible for such effect is yet to be investigated, although more EMMPRIN containing EVs were released in response to Ivabradine, and EMMPRIN containing cardiac EVs inhibited cell death and induced cell migration and proliferation of H9c2 cardiac derived cell cultures, accordingly with EMMPRIN-induced MMP-activation as in EMMPRIN-containing EVs from cancer cells [12,13,14].

The presence of EMMPRIN as a cargo component in microvesicles was associated to tumor cell invasion in several types of cancer [12,13,14], and in fibroblast uterine cells for endometrial remodeling [26]. In most cases, the role of EMMPRIN containing microvesicles includes cell migration and/or proliferation [27]. This is the first time showing that EMMPRIN-containing cardiac MVs is induced by Ivabradine. The relevance of this findings were assayed in cultured cardiac derived H9c2 cell monolayers, in which EMMPRIN Cardiac MVs released from pigs by day 7 after IR significantly reduced apoptosis, while on the opposite, cell migration and proliferation were both increased, concluding that Ivabradine at least, induced cardiac protection by releasing specific MVs, preventing cardiac cell necrosis. Extensive research in the composition of Ivabradine induced cardiac MVs may have a clinical implication stopping short term cardiac cell necrosis after infarction.

Cardiac regeneration is a very complex and tightly orchestrated series of events, mostly still unknown, in which cell migration may play a key role. Cardiac stem cells and cardiac side population cells have been tested in animal models of cardiac injury, including myocardial infarction [28]. H9c2 cell cultures, are mononucleated cells isolated from rat ventricular heart. H9c2 is a subclone of the original clonal cell line derived from embryonic BDIX rat heart tissue [29], isolated thirteen days after fecundation. Depending on the culture conditions, H9c2 cells remain in two differentiation stages: embryonic-like cells, already committed but still undifferentiated into cardiomyocytes, and in response to serum deprivation plus incubation with trans-retinoic acid, H9c2 become elongated, multinucleated and differentiated into cardiac like cells [30]. Here we found that EMMPRIN containing cardiac MVs isolated 7 days after CS from the plasma of pigs treated with Ivabradine, may induce H9c2 (cardiac embryonic undifferentiated) cell migration and proliferation, suggesting that, as in cancer cells, vehiculated EMMPRIN may play a critical role in cardiac cell migration through MMP activation, which is supported by the main composition of EMMPRIN found in cardiac MVs, as highly glycosylated, required for MMP activation.

The data presented in this work describe a new mechanism promoted by Ivabradine in the protection of the heart against the damage caused by IR, by at least inducing the release of EMMPRIN containing cardiac MVs. Further in vivo studies will be crucial to consider Ivabradine as a suitable tool for the treatment these and other cardiovascular diseases in which cell migration and proliferation could be a critical step for healing.

### Limitations

These data were obtained in a porcine model of IR. Increasing sample sizes, and a first human study will be the next steps for further validation. We cannot exclude that at least some of the HCN4+ microvesicles may have a neuronal origin, since HCN channels are also expressed in the peripheral and CNS. However, no HCN4+ MVs have been reported so far.

## 4. Materials and Methods

Animal procedures were performed in the Experimental Surgery Department of the Hospital Universitario La Paz (Madrid, Spain). The investigation conforms to the Guide for the Care and Use of Laboratory Animals published by the U.S. National Institutes of Health (NIH Publication No. 85-23, revised 1985), and the Animal Welfare Ethics Committee and complied with the EU Directive on experimental animals (63/2010 EU) and related Spanish legislation (RD 53/2013), PROEX 365-15.

### 4.1. Cardiac Ischemia/Reperfusion

Yorkshire female pigs (37.8 ± 5.2 kg) were subjected to cardiac ischemia/reperfusion as previously described [11].

### 4.2. H9c2 Cell Culture

H9c2 cells were grown in Dulbecco’s modified Eagle’s medium (DMEM) supplemented with 10% fetal bovine serum (FBS) from Sigma Aldrich (Saint Louis, MO, USA), 50 mg/mL of penicillin and 50 mg/mL streptomycin (Invitrogen, Carlsbad, CA, USA), and incubated at 37 °C in humidified atmosphere of 5% CO_2_ and 95% oxygen.

### 4.3. Extracellular Vesicles Isolation

To isolate plasma MVs, 5 mL of whole blood was collected at the times indicated and serial rounds of centrifugation were performed. Initially, the blood was centrifuged at 1200× *g* for 10 min at room temperature. The plasma was transferred to a sterile centrifuge tube and centrifuged at 2000× *g* for 30 min at 4 °C in order to remove cell debris and apoptotic bodies. The supernatant was transferred to another tube and centrifuged at 16,000× *g* for 30 min to concentrate the MVs, as described by the International Society of Thrombosis and Haemostasis [31]. The supernatant was discarded, and the pellet was resuspended in 500 µL of double-filtered PBS to obtain the final MV-enriched sample.

From now on, the EVs will be referred in the text as MVs, since they precipitated at 16,000× *g* and as will show in the results, as part of the cargo components, proteins of more than 100 kDa in size were detected. However other particles including apoptotic bodies and/or protein aggregates might be present in the samples, as well.

### 4.4. Microvesicles Characterization by Flow Cytometry

MVs were characterized following the guidelines of the International Society for Extracellular Vesicles [32], by using flow cytometry (FACS Calibur (BD Biosciences with CXP software) for quantification and membrane markers (see below), as well as confocal microscopy for size control (MVs ranged in size between 0.3 μm to 1.2 μm).

MVs were estimated with the aid of the fluorescence emission and size, considering those events with a diameter between 0.22 μm and 1.35 µm using fluorescent beads from the Flow Citometry Sub-Micron Size Reference Kit, from Thermo Fisher (Waltham, MA, USA). Events below 0.22 μm were considered background and subsequently discarded, as well those above 1.35 μm, considered apototic bodies [33].

MVs were also characterized by their protein composition. In particular, MVs were incubated with the following fluorescein isothiocyanate-conjugate (FITC) labeled monoclonal antibodies: anti-CD31 (BD Biosciences, Franklin Lakes, NJ, USA), anti CD41 (Stemcell technologies, Vancouver, BC, Canada), and anti-HCN4 (Santa Cruz Biotechnology (Dallas, TX, USA), with phycoerythrin-annexin A5 (BD Biosciences, Franklin Lakes, NJ, USA), in annexin A5-biding buffer (10 mM HEPES, 7.4 pH, 140 mM NaCl, 2.5 mM CaCl2). In addition, double binding as also performed by incubating MVs with the antibodies mentioned above and Alexa-Fluor 647 anti-EMMPRIN (Santa Cruz Biotechnology (Dallas, TX, USA). Equal amounts of calibration beads were incubated to quantify events per microliter. Exclusion of apoptotic bodies were also analyzed by incubating DNA isolated MVs with acridine orange (Invitrogen), as described [34].

### 4.5. Cell Cycle Phase Assay in H9c2

H9c2 MVs were isolated from the culture medium following the same procedure described above. 24 h prior to collection, cells were cultured in 0% FBS DMEM to avoid Serum MVs contamination. Briefly, culture supernatants were serially centrifuged (15 min at 1200× *g* to remove cell debris, and 30 min at 16,000× *g* to concentrate MVs).

Cells were incubated for 24 h with 10^7^ HCN4+/EMMPRIN+ MVs and detached with trypsin. Subsequently, cells were washed and fixated with cold 70% ethanol. Then, cells were resuspended in 500 µL of double-filtered PBS and stained with propidium iodide. To avoid the presence of RNA, RNAse A was added, and then the cell cycle phase was analyzed in the flow cytometer FACS Calibur, and the plots were analyzed.

### 4.6. Wound Healing Assay

H9c2 cells were grown in six-well plates, and a straight incision was made on the monolayer. Cell movement was monitored over a time course by microscopy and calculated by determining the area unoccupied by cells between 0 and 24 h. Images were analyzed using ImageJ software (version 1.53 d).

### 4.7. Protein Expression Determination by Immunoblot

Immunoblot was performed as described [16].

### 4.8. Statistical Analysis

All data were analyzed in a statistical software package (SPSS 22.0, SPSS Inc., Chicago, IL, USA). All values are given as mean ± S.D. Significance is reported at the 5% level. Whenever comparisons were made with a common control, significance of differences was tested by analysis of variance followed by Dunnett’s modification of the *t* test.

## Abbreviations

IR	Ischemia/reperfusion
AMI	Acute Myocardial Infarction
MV	Microvesicle
EMMPRIN	Extracellular Matrix Metalloproteinase Inducer
